# Metaverse and mental health, what about the future?

**DOI:** 10.1192/j.eurpsy.2023.1171

**Published:** 2023-07-19

**Authors:** A. M. Matas Ochoa, S. Rubio Corgo, I. Durán Cristóbal

**Affiliations:** Psychiatry, Infanta Leonor University Hospital, MADRID, Spain

## Abstract

**Introduction:**

The metaverse is a digital world created using different technologies like virtual reality (VR), augmented reality (AR), cryptocurrency and the internet.

Interest in the metaverse has grown in recent months in different fields and it could have potential application in the treatment of mental health disorders.

**Objectives:**

To gain a better understanding of metaverse and to explore its possible applications on mental health.

**Methods:**

Review of recent literature about the implications of the metaverse users in mental health.

**Results:**

Metaverse is a virtual universe where people can interact with other users, objects, and environments personifying an avatar. VR, AR and mixed reality (MR) have been used in the treatment and diagnosis of various mental health disorders for last years.

Attention deficit hyperactivity disorder, eating disorders, anxiety, phobias and post-traumatic stress disorder have been already benefited from VR. Also, there are results to treat persecutory delusions in psychosis. On the other hand, we know that to spend a significant amount of time playing 3D immersive games and using social media, could lead to insecurity, anxiety, depression and behavioural addiction.

The lack of evidence and these risks could be limitations to implement Metaverse for the therapeutic management of mental health.

Many companies have already started to develop virtual mental health clinics with mental health professionals serving patients in real time, some spaces have already offer group therapy sessions. Other immersive spaces have also been created for practising mindfulness, meditation, or yoga.

**Conclusions:**

The new technologys have changed the way that we socialise, work, and interact, even the way that we receive medical treatment. The metaverse could prove useful in the management of the mental health disorders that have already benefited from VR, but at the same time we could potentially lead to the worsening of others.

**Disclosure of Interest:**

None Declared

